# Outcomes in Patients with Long COVID-19 One Year After their Discharge from Intensive Care Units

**DOI:** 10.7759/cureus.81739

**Published:** 2025-04-05

**Authors:** Talarisree Chandrasekhar, Calerappa Ravishankar, Anke Geethanjali, Talari Lahari

**Affiliations:** 1 Department of Critical Care Medicine, Krishna Institute of Medical Sciences Saveera Hospital, Anantapur, IND; 2 Department of Microbiology, Krishna Institute of Medical Sciences Saveera Hospital, Anantapur, IND; 3 Department of Ophthalmology, Krishna Institute of Medical Sciences Saveera Hospital, Anantapur, IND

**Keywords:** clinical manifestations, follow up, one-year, sars cov-2, survivors

## Abstract

Introduction

Survivors of prolonged severe COVID-19 who are treated at ICUs are at risk for physical and psychological complications, including lung injury and multi-organ dysfunction. As the number of survivors of severe COVID-19 increases, it is necessary to understand the trajectory of the disease and the patient care needed after discharge from the ICU. This study tries to efficiently assess the long-term clinical sequelae among patients with prolonged severe COVID-19 who were admitted to the ICU, one year after their discharge. The parameters tested included the chronic obstructive pulmonary disease assessment test (CAT) score, pulmonary function tests, and laboratory data.

Materials and methods

The study population included 454 patients who were followed up one year after surviving ICU admission for severe COVID-19. All the patients who presented with signs and symptoms to the hospital were examined further. They underwent the necessary investigations, assessments, and systemic examinations. The results of all the laboratory and radiological investigations were reviewed. During the SARS-CoV-2 pandemic, all the patient details were entered into a hospital information management system from which the data was retrieved. Mean with standard deviation (SD) or median or interquartile ranges (IQR) were used to assess the continuous variables, whereas numbers and percentages were used for categorical variables. Statistical significance was calculated by the Chi-square test.

Results

The median age of the study population was 64 (IQR 57-74) years and 64.7% (294/454) were male patients. The median follow-up time was 367 days. During the follow-up period, 14.9% (68/454) of the patients were readmitted to the ICU. The mean length of hospital stay was 12 days (IQR 8-20 days). Among the readmitted patients (n=68), 17.6% (12/68) were on mechanical ventilation and the remaining 82.3% (56/68) received oxygen therapy. One patient underwent extracorporeal membrane oxygenation. The hospital mortality rate observed among these ICU survivors was 10.2%. The Health-Related Quality of Life (HRQOL) score at baseline i.e. before the ICU admission (52.5 (SD, 9.2); p<0.001) was better than that observed at the one-year follow-up (44.3 (SD, 9.5); p<0.001). Moreover, the clinical frailty scale and cognitive symptoms were significantly different at the follow up assessment versus the baseline (p<0.001). The proportion of patients with a grade of 0-2 on the Modified Medical Research Council (mMRC) dyspnea scale was almost similar at baseline and the one-year follow-up, whereas a breathlessness grade of 3-4 on the scale was observed in 39.8% of the study population.

Conclusion

The management of ICU survivors after severe COVID requires a multi-disciplinary approach. It includes preventive measures and rehabilitation services along with appropriate treatment strategies to relieve the residual symptoms.

## Introduction

Long COVID is the presence of post-acute sequelae after the COVID-19 infection, ranging from four to 12 weeks [1,2]. Many such patients suffer from persistent somatic symptoms or organ dysfunction for several weeks or months after the initial infection.

Survivors of long COVID are at increased risk of various outcomes such as diabetes, cardiovascular disease, neurological diseases, and kidney disease. Patients with prolonged severe COVID-19 illness treated in ICUs are at risk for physical and psychological complications, including lung injury and multi-organ dysfunction. Terms such as 'post-acute sequelae of SARS-CoV-2 infection (PASC)', 'Long COVID-19', and 'Post-acute COVID-19', have been used in this context [3].

Since the detection of severe acute respiratory syndrome coronavirus 2 (SARS-CoV-2) in China in December 2019, millions of people have been infected [4]. The highest number of ICU admissions were for COVID-19 patients as they needed mechanical ventilation and prolonged ICU stay [5]. Patients who are admitted to the ICU due to acute critical illness frequently suffer from post-intensive care syndrome (PICS), a collection of physical, cognitive, and psychological impairments [6], which can contribute to decreased health-related quality of life (HRQOL) [7]. It also increases the financial burden on the patients and their family members due to the prolonged hospital stay and their inability to return to work after discharge [8].

A systematic review and meta-analysis of 194 studies reported at least one unresolved symptom at four months in 45% of COVID-19 survivors, irrespective of their hospitalization status [9]. A prospective, multicenter, observational cohort study in the UK observed that five months after being infected with SARS-CoV-2, patients showed more abnormalities of the lungs, brain, and kidneys compared to controls without COVID-19 [10]. It is difficult to explain the differential diagnosis after assessing the clinical manifestations of the post COVID-19 condition [11]. Some short-term follow-up studies of COVID-19 patients who survived ICU admissions have observed greater physical, mental, and cognitive manifestations along with many respiratory signs and symptoms [12,13]. A few authors who conducted similar long-term follow-up studies on patients who survived ICU admissions after SARS-CoV-2 infection also reported similar clinical findings [14,15].

The number of patients who survive severe COVID-19 continues to increase. This mandates understanding the trajectory of severe COVID-19 and the need for patient care after hospital admission. This study tried to efficiently assess the long-term clinical sequelae along with the chronic obstructive pulmonary disease assessment test (CAT) score and other outcome measures among patients who survived for a year after ICU admission for long COVID.

This study aimed to evaluate the percentage of various clinical manifestations of severe COVID-19 in patients who survived ICU admission, one year after their discharge from the hospital. Parameters assessed during the follow-up included assessment of chest CT severity score, quality of life, clinical frailty, and cognitive score compared to the baseline.

## Materials and methods

Study design and patient population

This prospective longitudinal cohort study was conducted at Krishna Institute of Medical Sciences Saveera Hospital, Anantapur, from December 2021 to May 2023. All patients with laboratory-confirmed SARS-CoV-2 and later presenting with different clinical manifestations at our hospital were enrolled. Informed consent was obtained from the patients themselves or their legal guardians. All evaluations were carried out in accordance with relevant guidelines and regulations.

Inclusion and exclusion criteria

Adult patients (>18 years) admitted to the ICU during the first wave of COVID-19, those who presented with severe COVID-19, and those with SARS CoV-2 detected in the respiratory samples using real time polymerase chain reaction (RT-PCR). 

Exclusion criteria included patients who refused to participate and those who were lost to follow-up.

Data collection

During the SARS-CoV-2 pandemic, all the patient details were entered into a hospital information management system from which the data could be retrieved. Clinical and demographic data such as clinical features, demographic characteristics, comorbidities, relevant history, examination findings, Acute Physiology and Chronic Health Evaluation II (APACHE II), Sequential Organ Failure Assessment (SOFA), Charlson index, vital signs, antimicrobial therapy, complications, and outcomes were available in this electronic case report form (eCRF). All this data was entered into an Excel spreadsheet (Microsoft Corp., Redmond, WA, US). Follow-up data and patient details were collected prospectively during the period of the present study.

Follow-up protocol and assessment

The study population (n=454) who had undergone ICU admission were invited for a follow-up consultation after a year. Among them, patients presenting with symptoms were examined further by a team of experienced intensivists and an ICU nurse. Those with symptoms but not presenting to the hospital were contacted by the team via telephone, using pre-structured questionnaire which included clinical features, exercise intolerance, and CAT. A CAT score of at least 10 is recommended as the threshold for maintenance treatment in COPD [16]. A detailed medical history, including ICU stay and post-ICU recovery, was assessed. Patients underwent a thorough systemic examination, routine vital monitoring, and relevant diagnostic tests, including complete blood count, C-reactive protein levels, renal and liver function tests, pulmonary function tests, arterial blood gas (ABG) analysis, and chest CT imaging. Results of all the laboratory and radiological investigations were reviewed.

Outcome measure

WHO standards were used to define the disease severity of COVID-19 [17]. Mild COVID-19 cases often experience fever, cough, and fatigue. Moderate cases may have difficulty breathing or mild pneumonia, while severe cases may have severe pneumonia, other organ failure, and possible death.

A simplified acute physiology score (SAPS 2) was used within the first 24 hours of ICU stay on critically-ill patients to estimate the probability of mortality [18]. An assessment of the pulmonary status of all the study population was done by the modified Medical Research Council (mMRC) dyspnea scale. The clinical frailty score was measured and a score greater than or equal to five was considered 'frail' [19]. The Cognitive Failure Questionnaire (CFQ-14) was used to evaluate the cognitive symptoms. It consists of 14 questions with a five-point Likert scale which measures daily life cognitive failures ranging from 0 ‘never’ to 4 ‘very often’ and leads to diverse range from 0 to 100. The score of >43 was considered for the presence of cognitive symptoms [20]. CT scans were reported by experienced radiologists by blinding clinical data, focusing primarily on ground glass opacities, inter/intra lobular and irregular lines (reticulates), bronchiectasis, consolidations, and fibrosis.

Statistical analysis

Mean with standard deviation (SD) or median or Interquartile ranges (IQR) were used to assess the continuous variables and numbers and percentages were used for categorical variables. Statistical significance was calculated by the Chi-square test. A p-value of <0.05 was considered statistically significant.

## Results

A total of 835 COVID-19 patients were admitted to the ICU between December 2021 and May 2023. The mortality rate in this baseline population was 14.8% (124/835). Seventy-six patients were lost to follow up and the remaining patients (n=181) had moderate COVID-19. Patients categorized with severe COVID-19 (n=454) were included as the study population for this analysis and underwent a follow-up consultation one year after ICU discharge. The median age was 64 (IQR 57-74) years and 294 (64.7%) out of 454 were male patients. The median follow-up period was 367 days. During the one-year follow-up, 68 (14.9%) patients were readmitted to the ICU. Their mean length of hospital stay was 12 days (IQR 8-20 days). Among these patients (n=68), 12 (17.6%) were on mechanical ventilation and the remaining 56 (82.3%) received oxygen therapy. One patient underwent extracorporeal membrane oxygenation. The hospital mortality rate in the study population was 10.13% (46/454).

The most frequently noted clinical manifestations in the evaluated patients included physical symptoms such as fatigue (n=255; 56.1%), alopecia (n=185; 40.7%), post-traumatic stress disorder (n=214; 47.1%), and myalgia/arthralgia (n=188; 41.4%). Due to muscle weakness or fatigue, several patients experienced limitations in exercise tolerance (n=175; 38.5%) and 39.3% were unable to return to work even after one year. The majority of patients suffered from at least one comorbidity (n=186, 40.9%), predominantly hypertension, diabetes, and obesity (Table 1).

**Table 1:** Clinical manifestations observed in the study population (n=454)

Clinical manifestations	Number of patients	Percentage
Cognitive impairment	45	9.90%
Depression	142	31.20%
Reduced exercise tolerance	175	38.50%
Fever	38	8.37%
Cough	92	20.20%
Dyspnea	75	16.50%
Anxiety	156	34.30%
Chest pain	58	12.70%
Sore throat	22	4.80%
Fatigue	255	56.10%
Alopecia	185	40.70%
Anosmia	49	10.70%
Dysguesia	42	9.20%
Arthralgia/myalgia	188	41.40%
Post-traumatic stress disorder	214	47.10%
Inability to perform self-care independently	86	18.90%
Returned to work	276	60.70%
Coexisting diseases		
0	186	40.90%
1	102	22.40%
≥2	90	19.80%

The determination of CT severity score was very helpful for assessing the severity of pulmonary dysfunction. Pulmonary dysfunction was assessed as 'none to mild' if the score was ≤5, 'moderate' if the score was >5 to ≤10 and severe involvement of lungs was considered if the score was >10. More than three-quarters of the patient population (n=345; 75.9%) presented with none to mild pulmonary involvement one year after hospital discharge, whereas comparatively fewer patients presented with moderate (n=31; 6.8%) and 78 (17.1%) had severe (n=78; 17.1%) pulmonary dysfunction.

**Figure 1:**
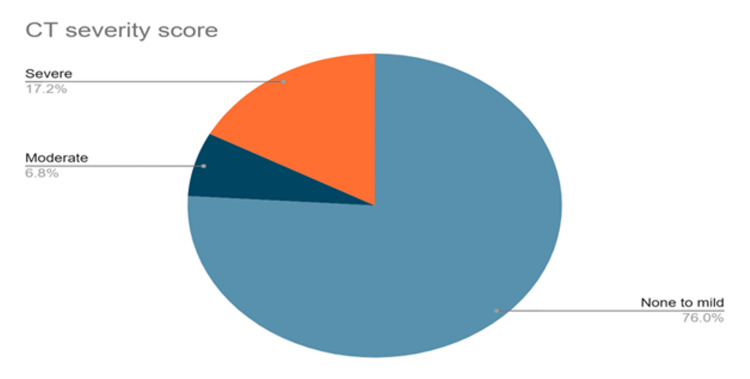
CT severity score in the study population (n=454)

Parameters such as HRQOL, clinical frailty score, and cognitive symptoms were assessed before the ICU admission during the pandemic (baseline) and again at the current follow-up (one year after hospital discharge). Baseline scores for HRQOL were better (52.5 (SD, 9.2); p<0.001) compared to the follow-up testing (44.3 (SD, 9.5); p<0.001). Similarly, the clinical frailty scale and cognitive symptoms were significantly different at the follow up assessment versus the baseline (p<0.001). On the other hand, a similar proportion of patients presented with the mMRC dyspnea grade of 0-2 at baseline and at the one year follow-up. A breathlessness grade of 3-4 on the mMRC scale was observed in many patients (n=181; 39.8%) at the one year follow-up (Table 2).

**Table 2:** Analysis of the health-related quality of life, clinical frailty, and cognitive scores in the study population (n=454) *Statistically significant PCS: physical component summary; MCS: mental component summary.

	Baseline	One year after ICU discharge	P value
Health-related Quality of Life			
Short form - 12 PCS - Mean (SD)	52.5 (9.2)	44.3 (9.5)	<0.001*
Short form - 12 MCS - Mean (SD)	55.4 (10.8)	43.2 (11.2)	<0.001*
Clinical frailty scale			
Score	2 (2-4)	3	<0.001*
Frail	24.5	8.6	<0.001*
Cognitive symptoms			
Cognitive failure questionnaire 14			
Score	20.3	28.5	<0.001*
Cognitive symptoms	7.4	24.2	<0.001*
mMRC scale			
Score 0-2	63.40%	60.20%	0.6628
Score 3-4	36.60%	39.80%	0.56

## Discussion

This study evaluated patients with severe COVID-19 one year after their discharge from the ICU. The median age of the study population was 64 (IQR 57-74) years and 64.7% (294/454) were male patients. The elderly and male patients predominantly experienced the post COVID-19 condition and its symptoms. These aspects are supported by other studies [21-23].

Among the physical symptoms manifested in this patient group, fatigue (56.1%), alopecia (40.7%), post-traumatic stress disorder (47.1%), and myalgia/arthralgia (41.4%) were the most frequently noted ones. Among the symptoms of long COVID, fatigue is the most common [24]. A study stated that almost two-thirds of patients examined exhibited new medical problems including fatigue, pain, and weakness [15]. Another study that documented COVID-19 symptoms one year after the infection reported anxiety (16%), depression (12%), and post-traumatic stress disorder (51%) in the patients [21]. Kumar et al. [25] reported dyspnea (18.6%), fatigue (10.5%), and mental health issues (9.3%) as the most common symptoms after discharge. After one year, the symptoms decreased to 11.9%, 6.6%, and 9%, respectively. Initially, patients reported common symptoms like limb weakness, body and joint pains, headache, vomiting, cough, fever, anosmia, chest pain, and ageusia, ranging from 1-6.7%, and at the one-year follow-up, their prevalence decreased to 0.1-4.5%. Rogers et al. [26] studied neuropsychiatric illness among severe COVID-19 patients and noted that adults infected with SARS-CoV-2 have double the risk of presenting with psychiatric and neuropsychiatric illnesses. They also reported high levels of depression, anxiety, and post-traumatic stress disorder. Patients who survived the COVID-19 infection most commonly experienced symptoms such as myalgia (21.5%), nerve pain (15.6%), and loss of smell (8.6%). Another study noted that the physical symptoms in ICU survivors admitted due to COVID-19 were lesser than those admitted due to non-COVID-19 causes [23]. There is confusion about whether the post COVID-19 syndrome is new and unique to COVID-19, as some symptoms are similar to chronic fatigue syndrome and other chronic illnesses associated with infections. In our study, muscle weakness or fatigue in the patients after hospital discharge led to limitations in exercise tolerance (38.5%) and inability to return to work even after a year (39.3%). Rousseau et al. [27] studied patients with post-COVID-19 syndrome three months after their discharge from the ICU and noted that almost 87% of them were unable to return to work.

During the one-year follow-up period, 14.9% (68/454) of our study population were admitted to the ICU. Their mean length of hospital stay was 12 days (IQR 8-20 days) and the hospital mortality rate was 10.2%. Neville et al. [22] surveyed post-COVID-19 ICU survivors at three and six months after discharge. The median follow-up was for 182 days and a mortality rate of 4.9% was observed before the receipt of the three-month surveys. Nearly 19.0% of patients reported continued symptoms and 1.6% were severely compromised due to COVID-19. In line with this study, most participants had at least one comorbid condition. Laurent et al. [21] observed that 51 out of 71 patients had less than seven days of hospital stay and four died after discharge from the hospital. Heesakkers et al. [23] studied COVID-19 and non-COVID-19 ICU survivors using a validated questionnaire and found that the APACHE IV scores (59.5 (SD, 15.9) vs 70.9 (SD 23.6), p = 0.001) were significantly lower and the ICU stay was significantly longer (median 14 (IQR 9 to 29) days vs 5 (IQR, 2 to 13) days, p <0.0001) in the former compared to the latter group.

In our study, 17.6% (12/68) of the readmitted patients were on mechanical ventilation and the remaining 82.3% (56/68) received oxygen therapy. One patient underwent extracorporeal membrane oxygenation. In another study, 9% of patients were readmitted six months after hospital discharge and 17% were on supplemental oxygen [22]. On the other hand, a survey conducted on ICU survivors one year after discharge noted that that not all patients needed supplemental oxygen and the median pulse oximetry at rest was 96% [21].

We noted that CT chest findings in 75.9% of patients showed none to mild pulmonary involvement after one year of hospital discharge, whereas 6.8% showed moderate, and 17.1% had severe pulmonary dysfunction. Lopez-Leon et al. [28] observed abnormalities in 35% of CT scans even 60-100 days after the initial presentation. A study in China that followed COVID-19 survivors three months after recovery, noted that nearly two-thirds of the non-critical patients had radiographic changes in their lungs [29]. Another one-year follow-up study reported abnormal chest CT findings in 72.3% of patients, bronchiectasis in 42.5%, ground glass opacities in 40.4%, and reticulates and nodules in 31.9%, along with anecdotal atelectasis and alveolar consolidation [21]. Comparisons in patients with severe COVID-19 with normal versus abnormal chest high-resolution CT (HRCT) at 12 months after discharge were done. Patients with abnormal radiographic changes at 12 months had increased length of hospital stay (p=0·027) and increased peak HRCT pneumonia scores (p<0·0001) [30]. Nearly 10% of long COVID-19 patients presented with abnormalities in pulmonary function tests, such as decreased diffusion capacity for carbon monoxide [28]. On estimation of lung function tests among COVID-19 survivors by Huang et al. [31] and Hui et al. [32], pulmonary dysfunction was noted in 53% and 28% of patients, respectively. After a one-year follow-up of patients with post COVID-19 condition, a few studies observed that 28% of the survivors had decreased pulmonary function and signs of pulmonary fibrosis [33,34].

The burden of persistent symptoms remains high in these patients. Huang et al. conducted a two-year follow up of patients hospitalized for COVID-19 and reported that 55% of them had at least one unresolved symptom [35]. Zhan et al. [36] did a prospective study on the three-year follow-up data of 1359 participants (54% men and 46% women). They noted that 54% of the study population presented with at least one clinical manifestation after hospital discharge at three years and also documented that there is a significant reduction in fatigue and muscle weakness from two years onwards. They also noted that the most common symptoms experienced by patients included sleep difficulties, fatigue or muscle weakness, and hair loss. The study also evaluated the association between age, sex, and disease severity. Age and disease severity were not found to be significant predictors, but after three years, female patients were more likely to have at least one symptom compared to male patients.

In our study, the proportion of patients with a grade of 0-2 on the mMRC dyspnea scale was almost similar at baseline and the one-year follow-up, whereas a breathlessness grade of 3-4 was observed in 39.8% of the patients. This is similar to Lauren et al. [21] who observed that 68.5% of ICU survivors had a classification of grade 2 as per the mMRC scale, whereas 16% patients had severe dyspnea (grade 3 as per the mMRC classification).

COVID-19 patients showed good HRQOL scores at baseline in our study (52.5 (SD, 9.2); p<0.001) as compared to one-year follow-up (44.3 (SD, 9.5); p<0.001). HRQOL outcome assessment by the Patient-Reported Outcomes Measurement Information System (PROMIS)-29 physical and mental health summary score was lower in the COVID-19 survivors when compared to normal US population [22]. Lauren et al. found that the scores of the median physical component summary (PCS) and mental component summary (MCS) were 44 and 45 respectively [21]. Physical limitations and quality of life were combined with muscle weakness in patients after ICU discharge [37,38]. Quality of life limitations persist over time and remain even one year after ICU discharge [39,40]. At the one-year follow-up, Heesakkers et al. [23] documented significantly higher scores in ICU survivors with COVID-19 compared to those without COVID-19 with regard to SF-12 PCS (45.2 (SD, 9.9) vs 40.4 (SD, 11.0); p <0.001) and MCS (48.5, (SD, 11.9) vs 44.4 (SD, 12.6); p<0.001) scores.

Recovery from COVID-19 is different for all patients. Some symptoms can improve quickly, while others last longer, irrespective of severity of the first SARS-CoV-2 infection . Long term sequelae can also be seen in individuals with a mild infection who may not need admission at hospitals [41]. Post COVID-19 condition may impact the patient's life or cause disability. Hence, such patients need to be evaluated further, and require adequate treatment and support.

Intensivists and physicians continue to explore the most effective treatments for long COVID, aiming to support patient recovery. Managing individuals with persistent COVID-19 symptoms remains a significant challenge. Greater insights are needed into the underlying causes of these lingering symptoms, the relationship between risk factors and the severity of post-COVID-19 syndromes, and potential therapeutic approaches. Moreover, there is no definitive link between the risk of long-term COVID-19 effects and factors such as age, sex, race, gender, comorbidities, viral load, or disease progression [42].

Strengths

This study aimed to help healthcare providers better understand the long-term effects of COVID-19 and evaluate the role of CT chest scans and HRQOL assessments in improving diagnostic accuracy and patient management. In this prospective, monocentric study, we examined the symptomatic sequelae of SARS-CoV-2 infection, the need for hospitalization, and the interventions required upon readmission one year after ICU discharge. By assessing cognitive questionnaires, 12-PCS and 12-MCS HRQOL scores, and the clinical frailty scale at baseline and after one year, we gained valuable insights into the health outcomes of these patients.

Limitations

This study did not evaluate the impact of COVID-19 reinfection or other viral agents causing pneumonia on pre-existing long COVID symptoms and related health outcomes. Additionally, risk stratification based on age, sex, comorbidities, genetic predisposition, immune function, and metabolic factors was not conducted, which could have helped identify significant risk factors associated with the symptoms of the post COVID-19 condition. The effect of COVID-19 vaccination on the health condition of the ICU survivors was not established as it required an in-depth study with adequate financial support. Further prospective cohort studies on these aspects along with inclusion of comparator populations are crucial in investigating the disease and its management strategies.

Diagnosing and managing post COVID-19 symptoms is a serious challenge due to the huge population that was exposed to SARS-CoV-2 pandemic. The symptom presentation and the difficulty in correlating them with underlying risk factors or other serious illnesses complicate both evaluation and treatment. Furthermore, pandemic-related factors such as social isolation, economic hardships, restricted healthcare access, and disruptions to daily life have added to the complexities of long-term COVID-19 care. 

## Conclusions

Management of patients with symptomatic long COVID needs a multi-disciplinary approach, including preventive measures, rehabilitation services, and appropriate treatment strategies to provide relief. Patients with these problems can be affected by other serious illnesses. In this study, the elderly and male patients were predominantly affected by post COVID-19 condition. Three years after the COVID-19 pandemic outbreak, treating physicians, epidemiologists, and scientists continue to evaluate the long-term effects on patients experiencing persistent symptoms in the post-COVID-19 period. Many patients face challenges in obtaining an alternative diagnosis. This study examined the clinical outcomes in patients with severe COVID-19 who survived ICU admission. The most commonly observed symptoms included fatigue, alopecia, post-traumatic stress disorder, and myalgia/arthralgia. Some of the patients in the study needed ICU admission and oxygen therapy, while for others, it was fatal. Even one year after hospital discharge, significant differences in the clinical frailty scale, cognitive symptoms, and HRQOL scores were observed in these patients compared to baseline assessments. While symptoms of post COVID-19 condition range from mild to severe, they can significantly impact daily functioning. This study aimed to elucidate the long-term effects of SARS-CoV-2 (Omicron wave) on the health status of COVID-19 survivors one year after discharge from the ICU. Continued research is essential to fully understand the lasting impact of this disease.
